# Does financialization inhibit enterprise innovation? Analysis of innovation behavior of Chinese enterprises based on evolutionary game

**DOI:** 10.1016/j.heliyon.2024.e35981

**Published:** 2024-08-10

**Authors:** Jianxin Tang, Rizhao Gong, Yun Shi, Huilin Wang, Meng Wang

**Affiliations:** a School of Business, Hunan University of Science and Technology, Xiangtan, 411201, China; b School of Intelligent Finance& Accounting Management, Guangdong University of Finance & Economics, Guangzhou, 510320, China; c Moray House School of Education, The University of Edinburgh, Edinburgh, EH8 8AQ, UK

**Keywords:** Financialization, Enterprise innovation, Investment returns, Demand scale, Market competition

## Abstract

Enterprise innovation remains a cornerstone of economic development, with the direct influence of financialization on enterprise innovation standing as a critical factor. In contrast with the existing research, this study constructs an evolutionary game model by utilizing the Cournot model to analyze the innovation behavior of enterprises, and analyzes the influence of financialization on enterprise innovation by incorporating investment returns, market competition, and demand scale into the research framework. In addition, this study selects the sample of the Chinese non-financial listed enterprises and using panel data for the period 2009 to 2021. Based on the findings from the empirical analysis, this study reveals that excessive financialization hinders innovation in Chinese enterprises. Additionally, an intermediary pathway involving 'financialization - investment returns - enterprise innovation' is identified as a transmission mechanism. The demand scale generated by innovation inversely correlates with the inhibitory effects of financialization on enterprise innovation behavior. Meanwhile, heightened market competition amplifies the inhibitory influence of financialization on innovation. This study provided valuable empirical evidence, facilitating the enhancement of enterprise innovation efficiency.

## 1 Introduction

Financialization refers to the process or trend in which corporate behavior is increasingly inclined towards financial investment. It means that the operation of enterprises is gradually influenced or controlled by institutional investment [1]. In 2015, the average profit margin of core business revenue for Chinese industrial enterprises was only 5.59 %. Among the Fortune 500 companies, this number plummeted to 2.3 %. In 2016, the average capital profit margin of 68 representative industrial enterprises in China soared to 14.65 %, while the average capital profit margin of commercial banks approached 15.00 %. The average investment return rate of real estate related financial assets exceeds 30 %, with a total of 767 listed companies investing 726.876 billion yuan. As of 2019, the proportion of financial assets allocated by physical enterprises has reached 89.26 % [2,3]. Financialization is like a deadly virus, which leads to hollowing out of enterprises by absorbing and strengthening internal economic resources [4], and has an inhibitory effect on the recovery of China's real economy. Combining with the urgent need for the transformation of China's current economic development, improving the innovation capabilities of Chinese enterprises is undoubtedly the key to solving the current development dilemma.

The innovation of Chinese enterprises is constrained by many internal and external factors. A noteworthy issue is how financialization will affect enterprise innovation behavior and whether it will further squeeze the innovation investment of enterprises? Given that enterprise innovation continues to be an important driving force for economic development and enhances a country's overall competitiveness [5], the direct impact of financialization on corporate innovation has received widespread attention [[6], [7], [8]]. In the context of technological innovation, financial market conditions can be explained as determining variables, and a company's innovation strategy is the dependent variable [9]. These studies elucidate the connection between financialization and enterprise innovation. It is widely believed that financialization plays a limiting role in innovation capability and strategy. However, most research results are derived from theoretical structures and lack empirical evidence. In addition, previous studies have failed to determine the impact of investment returns on financialization and enterprise innovation behavior, and have also overlooked the potential impact of market competition environment and demand scale on the relationship between the two.

Moreover, financialization and entrepreneurial innovation behavior are influenced by investment returns. The focus of financial investors on high returns and their demand for performance [10] has weakened the financial independence and independent decision-making ability of company management [11]. Therefore, different investment returns can lead to differences in enterprise innovation behavior decisions. However, how to characterize the impact of investment returns on financialization and enterprise innovation behavior is a new challenge.

Furthermore, the enterprise innovation behavior is related to the degree of market competition. Studies has debated the impact of market competition on enterprise innovation, with arguments for both a "promoting effect" and a "restraining effect". The impact of market competition on enterprise innovation has been a topic of debate among researchers. Some argue that it has a promoting effect, while others believe it has a restraining effect. Researchers who advocate for the "promoting effect" suggest that sufficient market competition helps to improve enterprise technological innovation activities, while enterprises lacking competition have low management efficiency and insufficient innovation motivation [12,13]. However, some researchers believe that the market competition will increase malicious competition among enterprises, reduce corporate profits, and force enterprises to be more inclined to cater to existing markets, which is not conducive to the choice of innovation behavior [[14], [15], [16]]. Therefore, the relationship between financialization and enterprise innovation behavior may be related to the degree of market competition, but the mechanism of this relationship needs further explanation.

In addition, the impact of financialization on corporate innovation behavior depends on different demand scales. In theory, an increase in market demand can provide financial support for expensive research and innovation activities. Optimistic demand expectations reduce the uncertainty of research and development efficiency, and the potential return on innovation increases with the increase in market scale [17,18]. These studies mainly focus on the financialization process and innovation in developed countries, such as Europe and the United States [19,20]. Therefore, considering the uniqueness of China's financialization process and enterprise innovation, they lack in-depth elaboration and analysis on how financialization affects the innovation of Chinese enterprises.

**Fig. 1:**
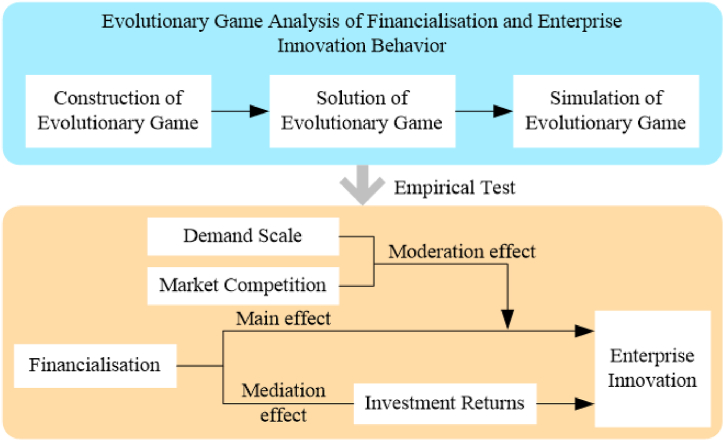
Flowchart of this study.

This study has made several contributions to the literature. Firstly, unlike traditional linear and static research paradigms, this study adopts the evolutionary game method to derive the dynamic evolution path of innovation behavior, and combines Chinese micro enterprise data for econometric research, improving the robustness of research conclusions. Secondly, it overcomes the limitations of existing literature on the assumption of homogeneous investment returns, and puts investment returns, market competition, and demand scale into a unified analytical framework, explaining the differential impact of investment returns on financialization and enterprise innovation behavior choices. Thirdly, the identification of the impact paths of different market competition environments and demand scales provides sufficient theoretical support for the government to formulate policies and enterprises to improve innovation efficiency. The process of this study is shown in Fig. 1.

## 2 Theoretical and literature background

### 2.1 Financialization and enterprise innovation

According to modern asset allocation theory, the goal of enterprises is profitability, while the objective of financial asset allocation is to invest funds reasonably across various assets, aiming to maximize returns while controlling risks. As the profitability of the real economy continues to decline, financial industry returns surpass those of real enterprises. When enterprises engage in financial asset allocation, they tend to avoid the real economy and instead enter the capital market, seeking profits through capital operations. As the proportion of financial asset allocation by enterprises continues to increase, it will inevitably encroach upon the funds intended for market expansion, scale expansion, and innovative research and development within the real economy. A substitution relationship is established between financial investment and innovation investment, whereby financial asset allocation may occupy resources originally allocated for innovation and research and development activities.

Enterprise innovation is a long-term, high-risk, and high investment effort that requires a significant amount of manpower and material resources. The development of finance and the degree of financialization have broadened the sources of funds, significantly improved financing capacity and efficiency, thereby promoting technological innovation [21,22]. With the advancement of financialization, the financial operation mode has undergone profound changes. Excessive financialization has changed the production pattern, shifting economic activity from the industrial sector to the financial sector [[23], [24], [25]]. This weakens the foundation of enterprise technological innovation. However, when capital is limited, increased financial investment will reduce the capital available for technological innovation. Essentially, financialization and core business innovation are interdependent. When a company's main business profit decreases but the return on investment in financial assets significantly increases, short-sighted executives may prioritize short-term financial investments as a means to quickly increase profits [26]. Therefore, excessive financialization has consumed a significant portion of corporate innovation funds, and funds used for high-risk long-term R&D investments have been redirected, thereby suppressing innovation momentum. Excessive return on financial assets has a negative impact on enterprise innovation investment.

Hypothesis 1 Excessive financialization has a negative impact on enterprise innovation.

### 2.2 Mediation effect of investment returns

The theory of agency suggests the separation of ownership and control within enterprises, where agency relationships entail principals signing agreements with agents, granting them partial decision-making authority. Specifically concerning corporate investment, under the circumstance of separation of ownership and control, managers, in pursuit of profit maximization, may adopt decisions deviating from the maximization of enterprise value, such as engaging in inefficient speculative investment behaviors. In the context of corporate financial asset allocation, the agency problem manifests as managers, upon being entrusted with managerial authority, may prioritize their own compensation or short-term improvement of corporate performance.

From the perspective of research and development (R&D) investment, managers under the agency problem tend to engage in short-term arbitrage, compounded by the profit-maximizing nature of capital itself. This leads to a preference for financial asset allocation in investment decisions.

Their inclination towards speculative arbitrage in investment decisions, driven by short-term profit pursuit and neglect of long-term enterprise development, gradually deviates from the goal of maximizing enterprise value. Moreover, such profit-driven investment decisions by managers do not necessarily indicate that corporate financial asset allocation is at a Pareto-optimal state. Instead, in this scenario, corporate financial asset allocation is considered passive investment, progressively diverging from the goal of shareholder value maximization, potentially exacerbating the agency problem.

If financial asset allocation brings about exceptionally high returns in the short term and improves corporate performance, managers' compensation is likely to increase. Under the agency problem, managers are more inclined towards enhancing corporate performance and their own compensation in the short term, rather than engaging in risky, slow-return, and long-cycle technological innovation investments. Consequently, they tend to favor increasing holdings of financial assets in investment decisions, driven by speculative arbitrage motives facilitated by the agency problem.

While an increase in firms' financial assets can enhance investment returns, alleviate financing constraints, accumulate retained earnings, and promote innovative investments, such an emphasis on financial investment returns can lead to a diversion from the core business, and corporate innovation, characterized by long cycles and high uncertainties, may be neglected. Moreover, an excessive reliance on financial investment returns can significantly increase corporate leverage [27], heightening financial risk levels and necessitating high cash holdings. This leaves enterprises with insufficient resources for stable R&D investments. Additionally, a high level of financialization implies that enterprise financial investments extend beyond traditional assets such as bonds, stocks, and investment properties to potentially include shadow banking [28]. Inflated financial investment returns further undermine enterprise R&D investments. Therefore, a high level of financialization may not only squeeze R&D funds but also create a dependency on financial investment returns, deterring enterprises from investing in innovation and potentially increasing earnings volatility while reducing innovation investments.

Hypothesis 2 Investment return is an intermediary pathway for financialization to suppress enterprise innovation.

### 2.3 Moderation effects of demand scale and market competition

From the perspective of financing constraints, the financing constraint theory points out that due to information asymmetry, the cost of external financing for enterprises is often higher than internal financing. Specifically concerning technological innovation, when enterprises lack sufficient stable internal funding support for technological innovation activities, they often resort to external channels such as bank loans to obtain debt financing. If enterprises hold a considerable amount of financial assets motivated by speculative arbitrage, the book value of financial assets is subject to potential risks arising from market fluctuations, thus reducing their debt repayment capacity.

In China, bank credit plays a significant role in the external financing of enterprises. Therefore, if enterprises hold a high proportion of financial assets, the potential risk of the book value of assets may affect the credit decisions of banks. Banks, in granting loans, become more conservative and cautious. To mitigate credit risk, banks will strengthen loan screening, to some extent, increase the cost of credit or reduce credit issuance. This will lead to higher financing costs for enterprises, i.e., increased financing constraints. The dilemma of financing constraints will further impact the development of enterprises' core business and investment in technological innovation, ultimately resulting in a crowding-out effect on technological innovation output.

The mechanism of demand scale driving enterprise technological innovation includes the ability to increase sales revenue to provide financial support for expensive research and innovation activities [29]. Optimistic demand expectations reduce the uncertainty of R&D performance [30], while market scale increases the potential profitability of innovation [18]. Empirical evidence supports the demand driven scale theory, indicating that sales play a crucial role in promoting research and development. This demand pull effect varies among different companies [31] and is influenced by income equality [32].

In addition, scholars like Schumpeter argue that market concentration and monopolies drive enterprise innovation, while excessively competitive markets suppress innovation [33]. The concentrated market provides the necessary financial support and risk tolerance for research and development activities. Effective technological innovation can further strengthen a company's monopoly ability and obtain monopoly rent [34]. Empirical research also supports this viewpoint, evidence indicating that restricting competition promotes technological innovation and productivity [35], while import competition from China has a negative impact on patent applications by American manufacturing companies [36]. Besides, mergers and acquisitions can increase innovation incentives by reducing competition [37,38]. Further testing is required.

Hypothesis 3 Demand scale mitigates the inhibitory effect of financialization on enterprise innovation.

Hypothesis 4 Market competition intensifies the inhibitory effect of financialization on enterprise innovation.

## 3 Evolutionary game analysis

The dynamic competition theory suggests that a company's behavior is influenced by the strategic decisions of its competitors. When a company's behavior may affect the profitability of its competitors, it will respond. The decision-making of enterprises is usually based on the principle of maximizing profits. In the first round of decision-making game, if a company chooses innovation but the overall return is lower than the innovation return, it is unlikely that the company will continue to implement innovation strategies in the following rounds. Therefore, whether a company chooses innovation or financial investment mainly depends on the returns of these two investment methods, which directly affect the company's profits. In addition, the demand scale and market competition can also affect a company's innovation decisions, thereby affecting profitability and ultimately influencing innovation behavior. Therefore, the decision of enterprises to make financial investments or innovations is influenced by the combined effects of investment returns, demand scale, and market competition.

In order to elucidate the impact mechanism of financialization on enterprise innovation in dynamic competitive relationships, this study adopts the Cournot competition model to illustrate corporate decision-making in a competitive environment.

Assumption 1 In this dynamic competitive market, there are two firms, denoted as Firm 1 and Firm 2, both producing homogeneous products.

Assumption 2 The market demand function for the firms' products follows a linear form, where ' a' represents the demand scale for each firm's product (a > 2). The firms' strategy involves determining their output, denoted as '$q_{i}$'. The payoff is their respective profit, ' $\pi_{i}$', and the production cost is ' $C_{i}\left(q_{i}\right)=q_{i}$'.

The output quantity ' $q_{i}$' is determined as

(1) $$q_{i}=a-p_{1}-p_{2},i=1,2$$

The profit for each firm is calculated as

(2) $$\pi_{i}=p_{i}q_{i}-C_{i},i=1,2$$

According to Eqs. (1), (2), the total income of the firms when allocating their funds for financial investment (FI) and technological innovation (RD) is represented as

(3) $$U_{i}=p_{i}q_{i}-\beta C_{i}+R,i=1,2$$

Assumption 3 Both firms currently possess surplus funds in addition to production capital. They need to make decisions regarding the allocation of these funds, choosing between financial investment (FI) and technological innovation (RD). Financial investment yields an income 'R' (R≥0). Based on market competition, the technological innovation reduces production costs by a factor of '*β*' (0 < β < 1), and the gross profit margin is '1-*β*'.

Assumption 4 The probability that Firm 1 selects the financial investment or technological innovation strategy is represented as '*x*' and '1-*x*', respectively. Similarly, the probability that Firm 2 chooses the financial investment or technological innovation strategy is '*y*' and '1-*y*', respectively. The results in the game payoff matrix for Firm 1 and Firm 2 is demonstrated in Table 1.

**Table 1:** Enterprise game payment matrix.

		Firm 2	
		Financial investment(y)	Technological innovation(1-y)
Firm 1	Financial investment(x)	${\left(\alpha -2\right)}^{2}/9+R,{\left(\alpha -2\right)}^{2}/9+R$	${\left(\alpha -\beta -1\right)}^{2}/9+R,{\left(\alpha -\beta -1\right)}^{2}/9$
	Technological innovation(1-*x*)	${\left(\alpha -\beta -1\right)}^{2}/9,{\left(\alpha -\beta -1\right)}^{2}/9+R$	${\left(\alpha -2\beta \right)}^{2}/9,{\left(\alpha -2\beta \right)}^{2}/9$

### 3.1 Expected returns of game agents

The expected returns of the two firms are obtained from the matrix as follows:

The expected profit of firm 1 when it chooses *FI* is

(4) $$E_{11}=y\left[{\left(a-2\right)}^{2}/9\right]+\left(1-y\right)\left[{\left(a-\beta -1\right)}^{2}/9\right]+R$$

The expected profit of firm 1 when it chooses *RD* is

(5) $$E_{12}=y\left[{\left(a-\beta -1\right)}^{2}/9\right]+\left(1-y\right)\left[{\left(a-2\beta \right)}^{2}/9\right]$$

The average expected profit of firm 1 is

(6) $${\bar{E}}_{1}=xE_{11}+\left(1-x\right)E_{12}$$

According to Eqs. (4), (5), (6), the expected profit when firm 2 chooses *FI*, the expected profit when firm 2 chooses *RD* and the average expected profit are

(7) $$E_{21}=x\left[{\left(a-2\right)}^{2}/9\right]+\left(1-x\right)\left[{\left(a-\beta -1\right)}^{2}/9\right]+R$$

(8) $$E_{22}=x\left[{\left(a-\beta -1\right)}^{2}/9\right]+\left(1-x\right)\left[{\left(a-2\beta \right)}^{2}/9\right]$$

(9) $${\bar{E}}_{2}=yE_{21}+\left(1-y\right)E_{22}$$

### 3.2 Evolutionary stable strategy based on the replicator dynamics function

According to Eqs. (4), (5), (6), (7), (8), (9), the system of replicated dynamic equations are

(10) $$\left\{ \begin{array}{c} F_{1}\left(x\right)=\frac{dx}{dt}=x\left(1-x\right)\left(E_{11}-E_{12}\right)\\ F_{2}\left(y\right)=\frac{dy}{dt}=y\left(1-y\right)\left(E_{21}-E_{22}\right) \end{array}\right.$$

From the replicated dynamic equations are all 0, we can get the pure strategy equilibrium points of the system as (0,0), (1,0), (0,1), (1,1), and the mixed strategy equilibrium points $\left(x^{*},y^{*}\right)$ where

$$x^{*}=y^{*}=\frac{{\left(a-2\beta \right)}^{2}-{\left(a-\beta -1\right)}^{2}-9R}{{\left(a-2\right)}^{2}+{\left(a-2\beta \right)}^{2}-2{\left(a-\beta -1\right)}^{2}}$$

### 3.3 Analysis of stability

#### 3.3.1 The Jacobian matrix

The stability of the system is determined using the Lyapunov discriminant method, and the evolutionary stability strategy (ESS) for the system of differential equations can be obtained by local stability analysis of the Jacobian matrix. The Jacobi matrix of the system can be obtained by letting the replicated dynamic equations all be first order derivatives of x and y respectively:

(11) $$J=\left[\begin{array}{cc} \frac{\partial F_{1}}{\partial x} & \begin{array}{cc} & \frac{\partial F_{1}}{\partial y} \end{array}\\ \frac{\partial F_{2}}{\partial x} & \begin{array}{cc} & \frac{\partial F_{2}}{\partial y} \end{array} \end{array}\right]$$

where, $\frac{\partial F_{1}}{\partial x}=\left(1-2x\right)\left[y{\left(a-2\right)}^{2}/9+R+\left(1-2y\right){\left(a-\beta -1\right)}^{2}/9-\left(1-y\right){\left(a-2\beta \right)}^{2}/9\right]$,

$$\frac{{\partial F}_{1}}{\partial y}=x\left(1-x\right)\left[{\left(a-2\right)}^{2}/9-2{\left(a-\beta -1\right)}^{2}/9+{\left(a-2\beta \right)}^{2}/9\right]$$

$$\frac{{\partial F}_{2}}{\partial x}=y\left(1-y\right)\left[{\left(a-2\right)}^{2}/9-2{\left(a-\beta -1\right)}^{2}/9+{\left(a-2\beta \right)}^{2}/9\right]$$

$$\frac{{\partial F}_{2}}{\partial y}=\left(1-2y\right)\left[x{\left(a-2\right)}^{2}/9+R+\left(1-2x\right){\left(a-\beta -1\right)}^{2}/9-\left(1-x\right){\left(a-2\beta \right)}^{2}/9\right]$$

#### 3.3.2 Eigenvalues of the jacobian matrix

Substitute each equilibrium point into Eq. (11), and find the eigenvalue of Jacobian matrix at the equilibrium point, when the eigenvalue is negative, it means that the equilibrium point is asymptotically stable.The eigenvalues of Jacobian matrix at the four equilibrium points are shown in Table 2.

**Table 2:** Eigenvalues of Jacobian matrix at each equilibrium point.

Equilibrium point	Eigenvalue 1	Eigenvalue 2
(0,0)	${\left(a-\beta -1\right)}^{2}/9+R-{\left(a-2\beta \right)}^{2}/9$	${\left(a-\beta -1\right)}^{2}/9+R-{\left(a-2\beta \right)}^{2}/9$
(1,0)	${\left(a-2\right)}^{2}/9+R-{\left(a-\beta -1\right)}^{2}/9$	${\left(a-2\beta \right)}^{2}/9-R-{\left(a-\beta -1\right)}^{2}/9$
(0,1)	${\left(a-2\beta \right)}^{2}/9-R-{\left(a-\beta -1\right)}^{2}/9$	${\left(a-2\right)}^{2}/9+R-{\left(a-\beta -1\right)}^{2}/9$
(1,1)	${\left(a-\beta -1\right)}^{2}/9-R-{\left(a-2\right)}^{2}/9$	${\left(a-\beta -1\right)}^{2}/9-R-{\left(a-2\right)}^{2}/9$

#### 3.3.3 Eigenvalue positivity and negativity determination and stability analysis

As ${\left(a-2\beta \right)}^{2}>{\left(a-\beta -1\right)}^{2}>{\left(a-2\right)}^{2}$, it can be obtained by calculation:

$${\left(a-\beta -1\right)}^{2}/9-\frac{{\left(a-2\right)}^{2}}{9}<{\left(a-2\beta \right)}^{2}/9-{\left(a-\beta -1\right)}^{2}/9$$

For the equilibrium point (0,0), when ${\left(a-\beta -1\right)}^{2}/9+R-{\left(a-2\beta \right)}^{2}/9<0$ , both eigenvalues are negative. The equilibrium point (1,0) and the equilibrium point (0,1) are unstable points because the conditions ${\left(a-2\right)}^{2}/9+R-{\left(a-\beta -1\right)}^{2}/9<0$ and ${\left(a-2\beta \right)}^{2}/9-R-{\left(a-\beta -1\right)}^{2}/9<0$ cannot be satisfied at the same time. For the equilibrium point (1,1), both eigenvalues are negative when ${\left(a-\beta -1\right)}^{2}/9-R-{\left(a-2\right)}^{2}/9<0$. In summary, the asymptotic stability conditions of each equilibrium point can be shown in Table 3.

**Table 3:** Stability of each equilibrium point.

Equilibrium point	Asymptotically stable condition
(0,0)	$R<{\left(a-2\beta \right)}^{2}/9-{\left(a-\beta -1\right)}^{2}/9$
(1,0)	Unstable
(0,1)	Unstable
(1,1)	$R>{\left(a-\beta -1\right)}^{2}/9-{\left(a-2\right)}^{2}/9$

**Fig. 2:**
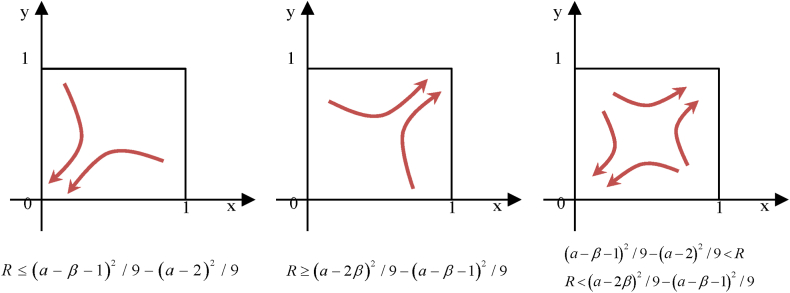
Evolutionary path of financialization and enterprise innovation behavior.

It can be seen from Fig. 2 that the point (0,0) was asymptotically stable when $R\leq {\left(a-\beta -1\right)}^{2}/9-{\left(a-2\right)}^{2}/9$ and (1,1) were asymptotically stable when $R\geq {\left(a-2\beta \right)}^{2}/9-{\left(a-\beta -1\right)}^{2}/9$. Both (0,0) and (1,1) were asymptotically stable when ${\left(a-\beta -1\right)}^{2}/9-{\left(a-2\right)}^{2}/9<R<{\left(a-2\beta \right)}^{2}/9-{\left(a-\beta -1\right)}^{2}/9$.

### 3.4 Numerical simulation of the evolutionary game model

Based on Matlab, this study verifies the conclusions of the game and compares the evolutionary paths of financial investment and innovation decision-making behavior of enterprises under different investment return scenarios, as well as the impact of different levels of market competition and demand scale.

The benchmark parameters are set as: a=3, β=0.5 and the initial values are set as $\left(x_{0}=0.4,y_{0}=0.5\right)$. The critical values are calculated as

$${\left(a-\beta -1\right)}^{2}/9-{\left(a-2\right)}^{2}/9=0.1389$$

$${\left(a-2\beta \right)}^{2}/9-{\left(a-\beta -1\right)}^{2}/9=0.1944$$

**Fig. 3:**
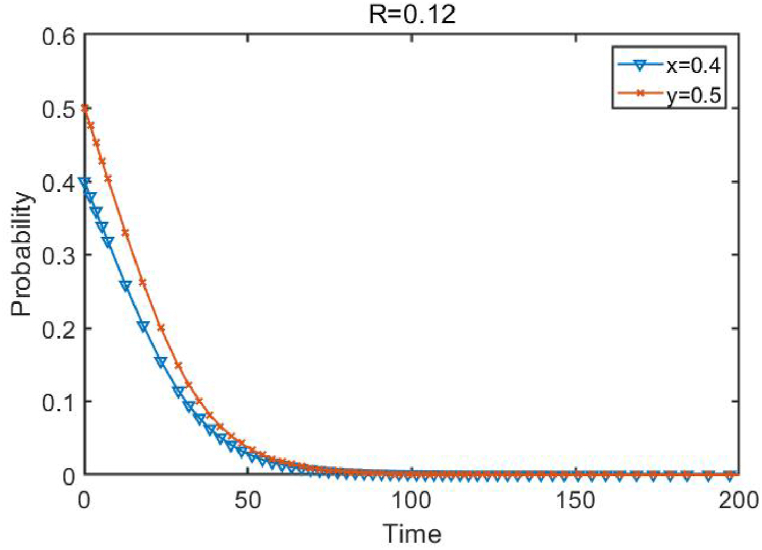
Evolutionary paths of different enterprise innovation behaviors.

When the conditions $R\leq {\left(a-\beta -1\right)}^{2}/9-{\left(a-2\right)}^{2}/9$ are met, *R* is set as 0.12. The simulation results are shown in Fig. 3. The initial probabilities for enterprises 1 and 2 to choose innovation are 0.4 and 0.5, respectively. When the investment return is below the critical point, it can be seen that the point (0,0) is asymptotically stable, and both enterprises have the motivation to choose innovation.

**when investment returns are small and**$R\leq {\left(a-\beta -1\right)}^{2}/9-{\left(a-2\right)}^{2}/9$.

**Fig. 4:**
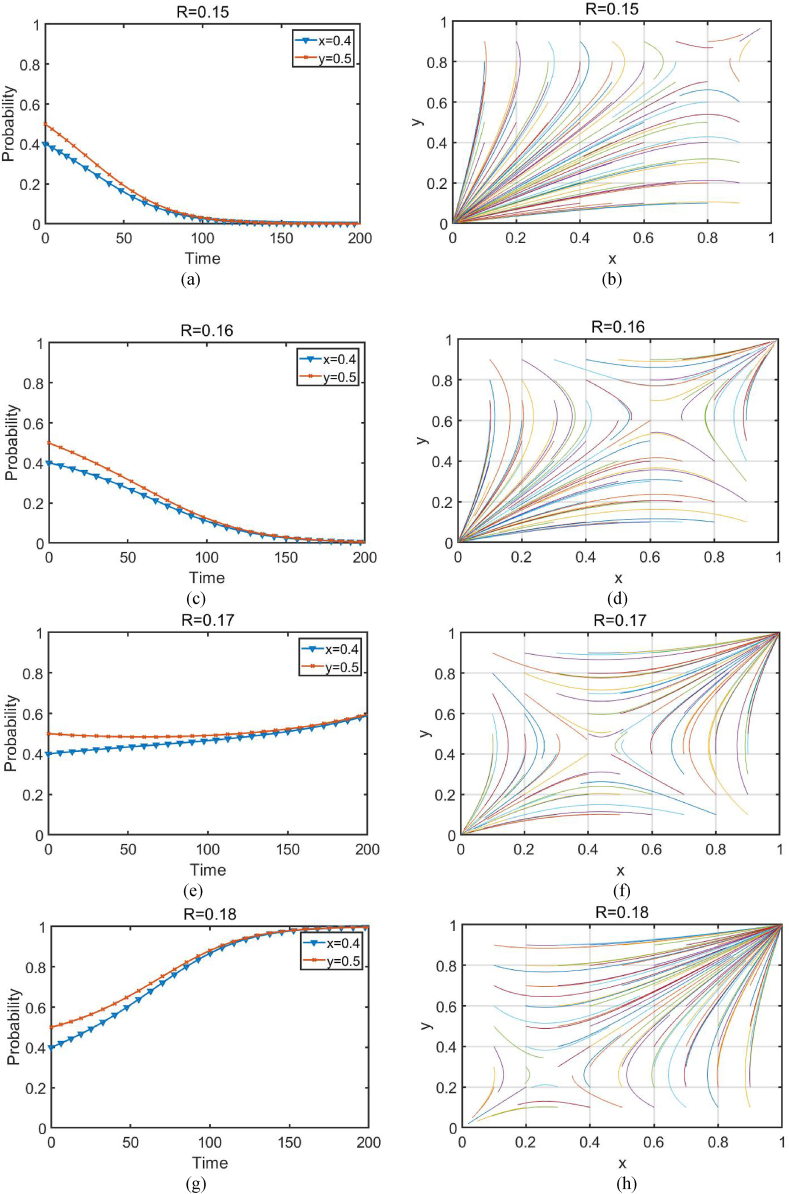
Dynamic evolution paths of different enteprise innovation behaviors.

When the conditions ${\left(a-\beta -1\right)}^{2}/9-{\left(a-2\right)}^{2}/9<R<{\left(a-2\beta \right)}^{2}/9-{\left(a-\beta -1\right)}^{2}/9$ are met, the benchmark parameters are set as: R={0.15,0.16,0.17,0.18} The simulation results are shown in Fig. 4. It can be seen that the point (0,0) and (1,1) are asymptotically stable point. The simulation results show that the smaller the investment return, the more likely the enterprise is to choose the (*RD*, *RD*) strategy, and the more investment return, the more likely the enterprise is to choose the (*FI*, *FI*) strategy.

**when**${\left(a-\beta -1\right)}^{2}/9-{\left(a-2\right)}^{2}/9<R<{\left(a-2\beta \right)}^{2}/9-{\left(a-\beta -1\right)}^{2}/9$. Note: (a), (c), (e) and (g) are evolution trajectory diagrams when R = 0.15, 0.16, 0.17 and 0.18, respectively. (b), (d), (f) and (h) are evolution phase diagrams when R = 0.15, 0.16, 0.17 and 0.18, respectively.

**Fig. 5:**
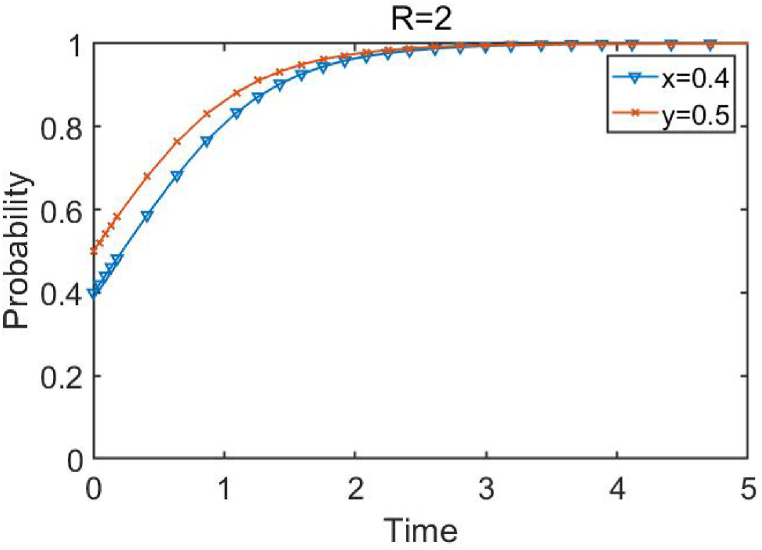
Dynamic evolution paths of Different enterprise innovation behaviors.

When the conditions $R\geq {\left(a-2\beta \right)}^{2}/9-{\left(a-\beta -1\right)}^{2}/9$ are met, *R* is set as 2. The simulation results are shown in Fig. 5. It can be seen that when *R* is above the critical point, (1,1) is an asymptotically stable point, and enterprises always choose not to innovate but to evolve towards financialization, regardless of the initial proportion of innovation. Research has shown that the higher the initial proportion, the faster the enterprise develops towards financialization, and the stronger the inhibitory effect of excessive financialization on enterprise innovation.

When investment returns are high and $R\geq {\left(a-2\beta \right)}^{2}/9-{\left(a-\beta -1\right)}^{2}/9$.

**Fig. 6:**
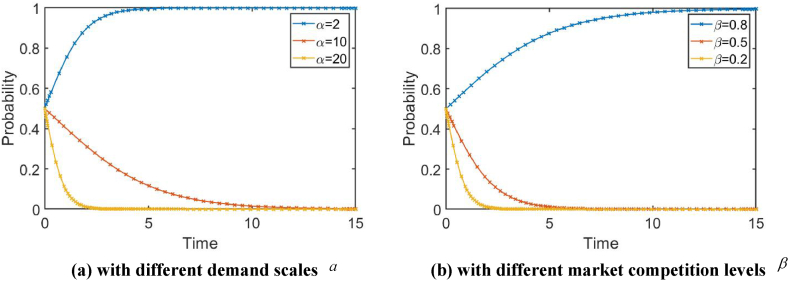
In the scenario where the investment return R is the same, the innovation behavior evolution path diagram of companies. Note: R = 1.2.

The simulation results at various α={2,10,20} and β={0.2,0.5,0.8} can be obtained when R=1.2 (in Fig. 6). It can be seen that the larger the demand scale, the less intense the market competition, and the greater the benefits of product innovation for enterprises. Enterprises are more likely to choose (*RD*, *RD*) strategies, indicating that the relationship between financialization and enterprise innovation is also influenced by the demand scale and market competition.

In summary, we can summarize that different enterprises not only compete but also exhibit a degree of complementarity in their production activities within an industry. A representative manifestation of this complementarity lies in enterprises' research and development (R&D) innovation activities. As enterprises invest substantial funds into R&D activities, they can enhance their product quality or reduce production costs, thereby gaining a competitive advantage within the industry. Moreover, the inherent externalities of innovation activities dictate that other enterprises within the industry also benefit significantly from the positive spillovers of research outcomes. In essence, an enterprise's R&D investment not only benefits itself through market competition but also extends benefits to other relevant enterprises in the industry. This inter-firm spillover characteristic of R&D activities within the industry implies that innovation activities confer substantial benefits to the overall industry, ultimately benefiting each individual enterprise.

However, in the pursuit of profit, enterprises often engage in behaviors characterized by short-termism. Given limited resources, if the return on investment in certain assets is high, enterprises tend to allocate more resources to those assets. With the excessive development of financialization, enterprises, in pursuit of immediate high returns, may opt to reduce or even eliminate funds allocated for technological innovation investment, redirecting them to purely financial investments. For individual enterprises, when the returns on financial investments are high, they may adopt a strategy of passive reliance on other enterprises to engage in innovative R&D investment, thereby benefiting from the spillover effects of others' innovation activities, thus making financial investment a strict ownership strategy for each enterprise. In this scenario, not only is the overall development of the industry compromised, but the final profits of each enterprise also decline significantly, resulting in a typical lose-lose outcome. The short-term rational choices of each enterprise ultimately lead to long-term irrational outcomes for the entire industry.

Furthermore, with the excessive development of financialization, the attractiveness of financial investment relative to technological innovation increases, exacerbating the crowding-out effect on funds for enterprise technological innovation. In circumstances where market demand scale is relatively high, market competition is relatively low, and enterprise R&D efficiency and returns are high, the inhibitory effect of financialization on enterprise innovation and R&D becomes more pronounced. In conclusion, excessive financialization inhibits enterprise innovation and R&D activities, diverting resources to pure financial investments rather than enhancing competitive advantages through R&D. Due to the complementarity of production activities and the positive externalities of R&D activities among different enterprises within the industry, individual enterprises engaging in free-riding behaviors ultimately lead to a sustained decrease in overall innovation and R&D in the industry. This further reinforces enterprises' willingness to venture into the financial investment domain, thus trapping the entire industry in a vicious cycle akin to a "prisoner's dilemma" [39].

## 4 Empirical analysis

In order to further verify the influence of financialization on corporate innovation decision-making behaviour, when the return on investment R is inflated, with different demand scale and market competition, this paper takes corporate innovation as the dependent variable and financialization as the independent variable, conducting empirical analyses.

### 4.1 Sample size and sources

Considering the availability and authenticity of data, this paper selects A-share listed companies in China's Shanghai and Shenzhen cities during 2009–2021 as the research sample, excludes the data of financial enterprises such as real estate, finance and insurance, cleaning up and deleting the samples of ST, IPO, LEV>1 and missing data in the current year, which results in 22,343 observations. The financial data used in the regression are mainly from CSMAR and WIND databases. Extreme values of the data are winsorised to reduce adverse effects. Table 1 reports the descriptive and correlation statistics of the variables.

### 4.2 Models and variables

#### 4.2.1 Empirical models

In order to illustrate the influence of financialization on the innovation of firms, test the mediating path of "financialization - investment returns - enterprise innovation", verify whether demand scale and market competition play a moderating role on the inhibitory effect of financialization on corporate innovation, testing the transmission path of *Y* to *X*. This paper constructs the following model:

(12) $$Y_{it}=\alpha_{0}+\alpha_{1}X_{it}+\alpha_{j}Controls_{it}+\varepsilon_{it}$$

(13) $$Z_{it}=\beta_{0}+\beta_{1}X_{it}+\beta_{j}Controls_{it}+\tau_{it}$$

According to Eqs. (12), (13), we can obtain

(14) $$Y_{it}=\gamma_{0}+\gamma_{1}X_{it}+\gamma_{2}Z_{it}+\gamma_{j}Controls_{it}+\varphi_{it}$$

(15) $$Y_{it}=\delta_{0}+\delta_{1}X_{it}+\delta_{2}M_{it}+\delta_{3}M_{it}\times X_{it}+\delta_{j}Controls_{it}+\theta_{it}$$

#### 4.2.2 Definition of core variables

**Enterprise Innovation (*Y*):** In this study, we use *Y1* and *Y2* to measure innovation. The first method evaluates technological innovation activities from the perspective of innovation investment, including factors such as R&D expenditure and R&D personnel allocation [40]. The second method evaluates innovation from an output perspective, including indicators such as the number of patent applications, patent citation rate, new product sales, and innovation efficiency [[41], [42], [43]]. Considering the availability of data for Chinese enterprises and the scientificity of indicators, this study measures enterprise innovation by taking the proportion of annual R&D expenditure to total assets as *Y1*, and the total number of applications for invention patents, utility models, and design patents plus the natural logarithm of 1 as *Y2*.

**Financialization (*X*):** In order to measure the degree of financialization, this study draws on the relevant research of Orhangazi and Demir [44,45]. It more directly reflects the financing behavior of the operating departments of manufacturing enterprises. *X1* is defined as the ratio of financialized financial assets to total assets, and the optimal level of financialization obtained from the actual level of financialization is *X2*. The higher the value, the greater the likelihood of excessive financialization [46].

**Investment Return (*Z*):***Z* is a proxy variable representing the return on investment of financial assets. It is calculated as the natural logarithm of investment return, achieved by adding 1 to the investment return. Higher value and ratio mean that companies are more inclined to seek investment arbitrage and returns through financial investments.

**Demand Scale (*M1*):** There are various methods in literature to measure the demand scale, with indicators often focusing on micro level enterprise sales and revenue, as well as macro level GDP and total demand [31,47]. In this study, we adopt a micro level indicator of operating revenue to evaluate the demand scale of enterprises, denoted as *M1*.

**Market Competition (*M2*):** Market competition can be measured in various ways. Tang uses subjective evaluation data to measure the degree of market competition, and many studies rely on objective indicators such as industry concentration, number of competitors, market entry barriers, and the Lerner index. This study follows the literature of Correa, Ornaghi [48], and Aghion [49], and argues that a company's gross profit margin level better reflects its competitive environment in operation. The gross profit margin of each enterprise ($pr_{it}$) varies, which can more directly reflect the specific competitive pressure of an individual enterprise. No matter how many competitors there are in the industry, the higher the gross profit margin, the less competitive pressure the enterprise faces. The higher the value of this indicator, the lower the overall competitive intensity within the industry. The calculation method for this indicator is as follows: $pr_{it}=\left(OP_{it}-rK_{it}\right)/S_{it}$. This variable is represented as *M2* = 1-$pr_{it}$, which moderates the relationship between financialization and enterprise innovation.

**Control variables:** Empirical research also includes control variables for corporate finance, governance structure, and external governance.

### 4.3 Results

#### 4.3.1 Excessive financialization inhibits enterprise innovation

In Table 4, Panel A presents the statistical descriptions of the research variables. The mean of the degree of financialization (*X1*) for enterprises is 0.0571, with a median of 0.0315, indicating that the proportion of financial asset allocation by non-financial enterprises in China is not high. This conclusion is further supported by the mean of *X2*, which is −0.0049, with a median of −0.0083. The mean and median of enterprise innovation (*Y1*) are 0.0194 and 0.0171, respectively, suggesting that the current level of innovation investment by enterprises in China is insufficient. The mean and median of investment returns (*Z*) are 16.3900 and 16.4400, respectively, indicating that the average level of investment returns for enterprises in China is relatively high. The descriptive statistical values of demand scale (*M1*), market competition (*M2*), firm size (*Size*), leverage (*Lev*), firm performance (*ROA*), number of directors (*Board*), independent directors (*Indep*), CEO-chairman duality (*Dual*), top 1 % shareholding (*Top 1*), firm age (*Firmage*), and management expenses (*Mfee*) are all reasonably aligned.

**Table 4:** Descriptive statistics and correlation matrix.

Panel A:	N	Mean	Sd	Min	P25	P50	P75	Max
Y1	22343	0.0194	0.0171	0.0000	0.0054	0.0171	0.0281	0.1030
Y2	22343	2.7560	1.6610	0.0000	1.6090	2.8900	3.9120	6.9600
X1	22343	0.0571	0.0710	0.0000	0.0078	0.0315	0.0800	0.5070
X2	22343	−0.0049	0.0397	−0.1300	−0.0193	−0.0083	0.0066	0.2050
M1	22343	21.6400	1.4000	18.8400	20.6500	21.4700	22.4600	25.7600
M2	22343	1.0400	0.0287	1.0060	1.0220	1.0320	1.0500	1.2000
Z	16786	16.3900	2.1850	3.0820	15.0900	16.4400	17.8200	21.3200
Size	22343	22.2500	1.2420	20.0300	21.3700	22.0700	22.9500	26.1600
Lev	22343	0.4260	0.1930	0.0593	0.2750	0.4240	0.5710	0.8590
ROA	22343	0.0408	0.0596	−0.2170	0.0144	0.0380	0.0693	0.2200
Board	22343	2.1340	0.1970	1.6090	1.9460	2.1970	2.1970	2.7080
Indep	22343	0.3750	0.0537	0.3330	0.3330	0.3570	0.4290	0.5710
Dual	22343	0.2670	0.4420	0.0000	0.0000	0.0000	1.0000	1.0000
Top1	22343	0.3410	0.1460	0.0845	0.2270	0.3200	0.4380	0.7330
FirmAge	22343	2.8860	0.3290	1.7920	2.7080	2.9440	3.1350	3.4970
Mfee	22343	0.0842	0.0590	0.0083	0.0441	0.0709	0.1070	0.3750

Panel B:	Y1	Y2	X1	X2	R1	R2	Z	Size	Lev	ROA	Board	Indep	Dual	Top1	FirmAge	Mfee
Y1	1															
Y2	0.317***	1														
X1	−0.082***	−0.103***	1													
X2	−0.023***	−0.031***	0.465***	1												
M1	−0.158***	0.363***	−0.016**	−0.016**	1											
M2	−0.181***	−0.169***	0.054***	0.029***	−0.390***	1										
Z	−0.173***	0.192***	0.324***	0.059***	0.522***	−0.00800	1									
Size	−0.224***	0.380***	0.045***	−0.00300	0.907***	−0.074***	0.591***	1								
Lev	−0.251***	0.141***	−0.083***	0.00400	0.519***	−0.100***	0.200***	0.497***	1							
ROA	0.153***	0.061***	0.00500	−0.00100	0.074***	−0.186***	0.070***	0.00600	−0.347***	1						
Board	−0.133***	0.033***	−0.036***	0.00100	0.250***	0.032***	0.140***	0.265***	0.157***	0.026***	1					
Indep	0.035***	0.032***	0.017**	0.00100	−0.00300	−0.00200	0.017**	0.0110	−0.00800	−0.024***	−0.532***	1				
Dual	0.151***	0.023***	−0.00100	0.011*	−0.159***	−0.00100	−0.098***	−0.155***	−0.115***	0.028***	−0.187***	0.108***	1			
Top1	−0.148***	−0.000	−0.049***	−0.00800	0.216***	−0.029***	0.110***	0.198***	0.058***	0.113***	0.044***	0.033***	−0.064***	1		
FirmAge	−0.079***	0.00900	0.174***	−0.021***	0.139***	−0.170***	0.122***	0.157***	0.116***	−0.081***	0.011*	−0.00500	−0.064***	−0.110***	1	
Mfee	0.251***	−0.081***	0.073***	0.029***	−0.534***	0.482***	−0.141***	−0.360***	−0.327***	−0.125***	−0.104***	0.041***	0.075***	−0.134***	−0.138***	1

Note:This table reports the descriptive statistics and correlation matrix of the variables used to test the hypothesis. The figures in the panel B represent the Pearson correlation coefficients.

***, ** and * represent statistical significance at the 1 %, 5 % and 10 % levels, respectively.

A preliminary judgment suggests that the degree of financialization, especially over-financialization, can influence the level of enterprise innovation. Therefore, the empirical part of the paper will examine this using *X1* and *X2*.

Panel B displays the Pearson correlation results of the research variables. It can be observed that the degree of financialization (*X1*) is negatively correlated with enterprise innovation (*Y1*) and (*Y2*), with correlation coefficients of −0.082 and −0.103, respectively. Similarly, *X2* is negatively correlated with enterprise innovation (*Y1*) and (*Y2*), with correlation coefficients of −0.023 and −0.031, respectively, indicating that financial asset allocation by enterprises can decrease their level of innovation and research and development.

Furthermore, the correlation coefficients between investment returns (*Z*), demand scale (*M1*), market competition (*M2*), and the degree of financialization (*X1* and *X2*) suggest that investment returns, demand scale, and market competition may affect the relationship between financialization and enterprise innovation. The correlation coefficients between firm size (*Size*), leverage (*Lev*), firm performance (*ROA*), number of directors (*Board*), independent directors (*Indep*), CEO-chairman duality (*Dual*), top 1 % shareholding (*Top 1*), firm age (*Firmage*), management expenses (*Mfee*) and the degree of financialization (*X1*) are 0.045, −0.083, 0.005, −0.036, 0.017, −0.001, −0.049, 0.174, and 0.073, respectively, indicating that there is a relatively low possibility of multicollinearity issues.

Table 5 shows the influence of financialization on enterprise innovation within non-financial listed firms. In this analysis, we utilized ordinary least squares (OLS) estimation in columns (1)–(2) and fixed effects (FE) estimation in columns (3)–(4) based on Eq. (12). The results reveal a significantly negative relationship between the degree of financialization and enterprise innovation among non-financial listed firms, with statistical significance at the 1 % level. These findings are obtained while controlling for various influences related to firm financials, internal dynamics, and external governance.

**Table 5:** Financialization and enterprise innovation (H1 testing).

	OLS		FE	
	(1)	(2)	(3)	(4)
	Y1	Y2	Y1	Y2
X1	−0.0155***	−0.9164***	−0.0079***	−0.6030***
	(-7.8725)	(-4.6759)	(-4.6401)	(-3.5498)
Size	−0.0005**	0.7164***	−0.0025***	0.5835***
	(-2.3696)	(38.9309)	(-7.4322)	(19.2811)
Lev	−0.0008	−0.0193	−0.0012	−0.2424**
	(-0.7030)	(-0.1812)	(-1.1596)	(-2.2687)
ROA	0.0479***	1.7576***	0.0116***	0.5254***
	(15.5458)	(7.0622)	(5.2756)	(2.8729)
Board	0.0015	0.0572	0.0012	0.0178
	(1.3807)	(0.5346)	(1.2523)	(0.1702)
Indep	−0.0012	−0.2566	−0.0043	−0.5021
	(-0.3457)	(-0.7077)	(-1.5269)	(-1.6254)
Dual	0.0009**	0.0660*	−0.0002	0.0641**
	(2.4170)	(1.9590)	(-0.5859)	(2.3337)
Top1	−0.0018	−0.1494	−0.0022	0.0861
	(-1.4282)	(-1.1903)	(-0.9848)	(0.4735)
FirmAge	−0.0030***	−0.1727***	−0.0049**	0.0260
	(-4.3012)	(-2.6949)	(-2.0149)	(0.1363)
Mfee	0.0512***	1.9811***	0.0098***	1.6267***
	(9.7487)	(6.1461)	(2.6950)	(5.8642)
Constant	0.0129**	−14.5299***	0.0704***	−11.1669***
	(2.3105)	(-30.0083)	(7.1125)	(-12.4977)
Year	Yes	Yes	Yes	Yes
Industry	Yes	Yes	Yes	Yes
*N*	22343	22343	22343	22343
Adj-R^2^	0.4567	0.4838	0.1368	0.2720

Note: This regression tests the effect of the financialization (X1) on enterprise innovation (Y1 and Y2). The t-values are presented in parentheses. ***, ** and * represent statistical significance at the 1 %, 5 % and 10 % levels, respectively.

In terms of economic significance, this outcome aligns with the expectations outlined in research hypothesis 1, suggesting that excessive financialization indeed crowds out enterprise innovation at this stage. Specifically, as the degree of financialization increases, the quality of enterprise innovation tends to deteriorate.

The enterprises tend to allocate their financial assets toward market arbitrage rather than channeling them into the high-quality development of enterprise innovation. While financial investments can generate certain returns and boost enterprise cash flow positively, the primary motive behind capital allocation becomes profit-seeking through capital arbitrage. Furthermore, financialization disrupts the structure of an enterprise's internal financing channels. Long-term investments in innovation and research and development activities are adversely affected by the crowding-out effect, as investment funds are diverted towards financial resource-mismatch activities, inhibiting investment in core business innovation. Additionally, financialization affects the internal governance structure of companies, influencing the incentives for innovation. It results in corporate cross-shareholding, institutional investor intervention, and executive equity incentive changes within corporate governance structures. This reduces the autonomy and control of corporate capital, directing capital allocation away from industrial development and towards high-return financial investments. Consequently, it affects the decision-making process for enterprise innovation and investment.

Over time, enterprise asset allocation behavior significantly deviates from the development of the core industry and innovation investments. Enterprises find themselves trapped in a "prisoner's dilemma" regarding high-quality innovation. If this trend persists, the real industry chain risks becoming "locked at the low end."

#### 4.3.2 Financialization influences firms' innovation through investment returns

Table 6 reports the empirical results of the "mediated transmission mechanism of financialization influencing enterprise innovation through investment returns" based on the sequential recursive model test of Eq. (14). The regression coefficients of Y1 and Y2 in Table 6 are both negative and significant in column (1)–(2), indicating that the main test of financialization inhibiting enterprise innovation still holds, and significantly positive in column (4)–(5), which shows that financialization improves enterprise’ investment returns. This paper focuses on the regression coefficient of investment return Z which is significantly negative after considering the intermediary variables. It can be inferred that investment return is a mediating variable that affects financialization and enterprise innovation behavior. Specifically, under resource constraints, the investment returns will influence the investment motivation of enterprises in the financialization process. Due to the "siphon effect" of high investment returns in financial assets, a large number of non-financial enterprises have abandoned diversified investments and high-quality development paths in their main businesses, preferring to arbitrage limited funds into financial assets and become professional "financial assets". Become a specialized "capital arbitrageurs" to reduce the investment of innovation resources, weakening enterprise innovation and research and development, and ultimately manifested as a "crowding out effect" to inhibit enterprise innovation.

**Table 6:** The mechanism of financialization's impact on enterprise innovation (H2 testing).

	Step 1		Step 2	Step 3	
	(1)	(2)	(3)	(4)	(5)
	Y1	Y2	Z	Y1	Y2
X1	−0.0088***	−0.5686***	4.9161***	−0.0083***	−0.5057***
	(-5.1882)	(-3.0763)	(16.6415)	(-4.9483)	(-2.7235)
Z				−0.0001*	−0.0128**
				(-1.7003)	(-2.0817)
Size	−0.0025***	0.5824***	0.8944***	−0.0025***	0.5939***
	(-6.9421)	(17.2307)	(17.3104)	(-6.7043)	(17.4020)
Lev	−0.0012	−0.1904	−0.2501	−0.0013	−0.1936
	(-1.0515)	(-1.4793)	(-1.3140)	(-1.0708)	(-1.5050)
ROA	0.0107***	0.4292*	3.2425***	0.0110***	0.4707**
	(4.5973)	(2.0369)	(8.8947)	(4.6647)	(2.2205)
Board	0.0012	0.0375	−0.0417	0.0012	0.0370
	(1.2230)	(0.3217)	(-0.2253)	(1.2196)	(0.3175)
Indep	−0.0046	−0.5390	0.4864	−0.0045	−0.5327
	(-1.6074)	(-1.5348)	(1.0225)	(-1.5926)	(-1.5171)
Dual	−0.0001	0.0644**	0.0170	−0.0001	0.0646**
	(-0.4266)	(2.1342)	(0.3402)	(-0.4219)	(2.1431)
Top1	−0.0011	−0.0366	−0.5097*	−0.0011	−0.0431
	(-0.4857)	(-0.1786)	(-1.7853)	(-0.5064)	(-0.2105)
FirmAge	−0.0057**	−0.1359	0.8687***	−0.0056**	−0.1248
	(-2.2645)	(-0.6137)	(2.8327)	(-2.2338)	(-0.5638)
Mfee	0.0065*	1.8355***	3.5037***	0.0068*	1.8803***
	(1.7397)	(5.8346)	(6.7126)	(1.8206)	(5.9850)
Constant	0.0736***	−10.9206***	−7.6072***	0.0729***	−11.0179***
	(6.6548)	(-11.0667)	(-5.3495)	(6.5978)	(-11.1759)
Year	Yes	Yes	Yes	Yes	Yes
Industry	Yes	Yes	Yes	Yes	Yes
*N*	16786	16786	16786	16786	16786
Adj-*R*^2^	0.1485	0.2681	0.1965	0.1488	0.2684

Note: This regression test the pathways through which financialization affects corporate innovation. The results indicate a suppressive effect of financialization on enterprise innovation, which is mediated through investment returns. The t-values are presented in parentheses. ***, ** and * represent statistical significance at the 1 %, 5 % sand 10 % levels, respectively.

#### 4.3.3 Demand scale and market competition have moderating effects

In columns (1)–(2) of Table 7, the results report in detail the relationship between financialization and enterprise innovation is affected by demand scale according to Eq. (15). The regression coefficients of *X1* are both significantly negative, and the regression coefficients of the interaction term M1*X1 are 0.003 and 0.3069 respectively, which are both significantly positive at 5 % level. The results meet the expectations of research hypothesis 3. A reasonable reason is that as demand scale gradually increases, more investment is made in technological innovation activities, and enterprises gradually accumulate the financial and human resources required for technological innovation. Technological innovation activities begin to become active in large enterprises, and technological innovation has a greater scale effect. In short, market demand can weaken the inhibitory effect of financialization on enterprise innovation.

**Table 7:** The effect of the financialization and enterprise innovation conditional on demand scale (H3 testing) and market competition (H4 testing).

	(1)	(2)	(3)	(4)
	Y1	Y2	Y1	Y2
X1	−0.0030*	−0.3667*	−0.0073***	−0.5657***
	(-1.7453)	(-1.9562)	(-4.3785)	(-3.3352)
M1	0.0095***	0.2545***		
	(22.2670)	(6.5071)		
M1*X1	0.0030**	0.3069**		
	(2.3654)	(2.4671)		
M2			−0.1165***	−4.3436***
			(-16.0346)	(-6.5209)
M2*X1			−0.0860*	−9.4329**
			(-1.8489)	(-2.0834)
Size	−0.0098***	0.3910***	−0.0012***	0.6282***
	(-20.9634)	(9.2733)	(-3.6840)	(20.2048)
Lev	−0.0042***	−0.3091***	−0.0029***	−0.2985***
	(-4.2417)	(-2.8678)	(-2.8330)	(-2.7721)
ROA	0.0036*	0.3150*	0.0059***	0.3242*
	(1.7519)	(1.6909)	(2.8144)	(1.7450)
Board	0.0005	−0.0031	0.0007	−0.0007
	(0.4806)	(-0.0296)	(0.7650)	(-0.0065)
Indep	−0.0060**	−0.5490*	−0.0053*	−0.5391*
	(-2.2048)	(-1.7863)	(-1.8898)	(-1.7516)
Dual	0.0000	0.0688**	−0.0002	0.0653**
	(0.0111)	(2.5160)	(-0.5274)	(2.3867)
Top1	−0.0023	0.0707	−0.0023	0.0748
	(-1.0605)	(0.3896)	(-1.0345)	(0.4122)
FirmAge	−0.0073***	−0.0356	−0.0066***	−0.0276
	(-3.1049)	(-0.1867)	(-2.7438)	(-0.1453)
Mfee	0.0493***	2.6959***	0.0381***	2.6931***
	(12.0129)	(8.2886)	(8.9286)	(8.1110)
Constant	0.0346***	−12.2320***	0.1695***	−7.4625***
	(3.5614)	(-13.6287)	(14.6857)	(-7.0969)
Year	Yes	Yes	Yes	Yes
Industry	Yes	Yes	Yes	Yes
N	22343	22343	22343	22343
Adj- R^2^	0.2099	0.2757	0.1725	0.2752

Note: This regression tests the effect of the financialization (X1) on enterprise innovation (Y1 and Y2) conditional on demand scale (M1) and market competition (M2). The t-values are presented in parentheses. ***, ** and * represent statistical significance at the 1 %, 5 % and 10 % levels, respectively.

The columns (3)–(4) of Table 7 consider the heterogeneity of market competition. In different levels of market competition, there are differences in the allocation of financial assets and innovation investment of enterprises. The results show that the regression coefficients of *X1* are both significantly negative at the 1 % level, and the regression coefficients of the interaction term M2*X1 are 0.086 and 9.4329, which are significant negative. From the above results, it is clear that excessive market competition reinforces the negative relationship between financialization and enterprise innovation. This result supports the hypothesis 4. In addition, this result also reveals that financialization has significantly reduced the innovation input and output of enterprises, while the degree of market competition further amplifies this negative impact. With the further increase of *M2* index value, the degree of market competition gradually rises to a higher level, and enterprises gradually lose market power and profitability. Investment expenditure on technological innovation activities begins to decline, and excessive competition is not conducive to technological innovation. Enterprises do not engage in substantial independent innovation and rely on technology spillovers to make innovation decisions converge in a poor lock-in state.

#### 4.3.4 Solution for endogeneity

The paper tackles the endogeneity concern by delving into the possibility of a bidirectional causality between financialization and corporate innovation. On one side of the argument, the advancement of corporate financialization could impede innovative endeavors. This occurs as financialization may prompt corporate managers and investors to overly prioritize short-term financial gains, consequently neglecting long-term innovation investments. Such shortsightedness risks compromising the future innovative potential of corporations. Conversely, corporations might prioritize long-term innovation over short-term profit maximization. This strategic shift entails reducing reliance on financialization mechanisms and channeling more resources into innovation pursuits. Consequently, this counteractive effect complicates accurately estimating the relationship between financialization and corporate innovation. To address these issues, we use lagged one period *X1*_*t-1*_ as instrumental variables, and conduct endogeneity tests using the two-stage least squares (2SLS) method [50]. Lagged variables, which are typically uninfluenced by current error terms, are utilized to circumvent contemporaneous endogeneity issues effectively. Furthermore, 2SLS tackles endogeneity concerns through a two-stage regression approach. The results are shown in Table 8. In the first stage, using lagged one period *X1* (*X1*_*t-1*_) as an instrumental variable, the regression shows a significantly positive coefficient for the instrumental variable, indicating a strong correlation between the instrumental variable and the explanatory variable *X1*. In the second stage of regression analysis, the coefficients of *Y1* and *Y2* are notably negative. Specifically, the coefficient of *X1*_*t-1*_ on *Y1* is −0.0164, significant at the 1 % level, while the coefficient of *X1*_*t-1*_ on *Y2* is −0.8183, significant at the 5 % level. These results are consistent with the baseline regression findings, further supporting the conclusions drawn in Hypothesis 1.

**Table 8:** Results of endogeneity test using 2SLS.

	First stage	Second stage	
		Y1	Y2
X1		−0.0164***	−0.8183**
		(-5.2694)	(-2.2249)
IV.X1t-1	0.4642***		
	(61.5374)		
Size	−0.0041***	−0.0025***	0.5799***
	(-4.5451)	(-14.7631)	(28.4009)
Lev	−0.0173***	−0.0019***	−0.2501***
	(-4.7841)	(-2.6694)	(-3.0433)
ROA	−0.0205***	0.0084***	0.5129***
	(-2.8895)	(6.2063)	(3.1943)
Board	0.0051	0.0014**	−0.0543
	(1.4841)	(2.1970)	(-0.6970)
Indep	0.0015	−0.0033*	−0.6423***
	(0.1441)	(-1.6762)	(-2.7721)
Dual	0.0016	−0.0002	0.0474**
	(1.5988)	(-0.7888)	(2.0355)
Top1	0.0020	−0.0036***	0.0130
	(0.3685)	(-3.5242)	(0.1068)
FirmAge	0.0309***	−0.0050***	−0.0165
	(4.9731)	(-4.1810)	(-0.1162)
Mfee	0.0300***	0.0073***	1.9486***
	(2.7653)	(3.5159)	(7.9323)
Year	Yes	Yes	Yes
N	18272	18081	18081
R2	0.291	0.108	0.240

The t-values are presented in parentheses. ***, ** and * represent statistical significance at the 1 %, 5 % and 10 % levels, respectively.

#### 4.3.5 Robustness test

To ensure the stability of the test results at the micro enterprise level, robustness testing will be conducted. Independent variable substitution (*Y1*, *Y2*) and dependent variable substitution (*X1*, *X2*) are used to better ensure the consistency of research test results and the reliability of conclusions. The consistency between the test results and the basic regression results can indicate that the empirical test results are robust and reliable.

## 5 Discussions

This study starts with an evolutionary game model and empirical analysis to analyze the impact mechanism of financialization on enterprises innovation. The following conclusions of this study are drawn.

The innovative behavior of enterprises is negatively affected by financialization. As investment returns increase, there is a corresponding stronger arbitrage motivation, which weakens the driving force of technological innovation and exacerbates negative inhibitory effects. In addition, the demand scale is a key factor affecting the relationship between financialization and enterprises innovation. As the demand scale increases, the likelihood of enterprises adopting innovation strategies decreases. This indicates that excessive financialization hinders the innovative behavior of enterprises and has a more significant inhibitory effect on companies operating within weaker demand scales. In addition, the level of market competition faced by enterprises significantly affects their innovation decisions. In a more competitive market, the rising cost of innovation makes financialization more attractive, thereby suppressing innovation.

## 6 Conclusions

This study suggests that companies should set long-term development goals, return to their main business, improve core competitiveness, focus on user needs and user experience, and innovate products and business models. Enterprises should continuously expand the scale of market demand and gradually achieve a positive transformation from competition driven to demand driven.

Based on the above conclusion, the following policy implications can be proposed.

Firstly, the government should strengthen financial supervision to prevent asset foam, use new methods such as digital technology to strengthen financial supervision [51], improve the efficiency of financial supervision, and crack down on financial speculation. Policy makers should adhere to the principle of "housing is for living, not for speculation", allowing real estate to return to its residential nature and squeeze speculative funds out of the real estate market. Financial institutions should strive to achieve high-quality supply of financial assets, accelerate the cultivation of multi-level capital markets, guide more funds to flow into the real economy, and provide financial support for the high-quality innovation and development of real enterprises.

Secondly, the government should focus on cultivating market mechanisms and enhancing market vitality. At the same time, attention should be paid to expanding the scale of demand and improving market efficiency. In order to achieve these goals, it is necessary to quickly carry out market-oriented basic price system reform, establish a mature market price mechanism, and revitalize the market. In addition, accelerating the protection of intellectual property rights to stimulate innovation and continuously stimulate consumer demand for high-quality products is a crucial step.

Thirdly, the government should actively optimize the market competition environment of the real economy. For companies with fierce market competition and severe product homogenization, the government should reduce direct subsidies to companies and encourage more companies to actively carry out innovation and product upgrades. For companies with low market competition and high product differentiation, more government subsidies and tax incentives should be allocated to support resources, encouraging companies to combine their own advantages and knowledge resources to provide more high-quality products and services to the market. In addition, encourage industry-leading companies to engage in mergers and acquisitions to increase industry concentration and profits, and provide sufficient profit margins for enterprise research and innovation activities.

Faced with the complex and ever-changing economic and political environment, we still need to be vigilant about the negative impact of financial speculation on enterprise innovation during the process of industrial structure upgrading. Although this study is based on empirical data from China, this conclusion also has implications for countries and regions with rapid development of financialization.

While this study delves into the adverse effects of financialization on enterprise innovation, it encounters limitations in both research scope and data availability. Despite employing econometric methods, the analysis overlooks the varying impacts of different financial assets and forms on innovation activities, indicating a promising avenue for future investigation. Furthermore, constrained by data availability, the study confines its analysis to Chinese data from 2009 to 2021, precluding examination of impact patterns amid low economic investment returns. Consequently, future research could broaden its scope through expansive data collection and deeper exploration of financialization's influence on innovation across diverse contexts. Additionally, integrating insights from game theory may further enrich our understanding of enterprise behavior in financialized environments, albeit facing challenges posed by data limitations, particularly in modeling competitive dynamics between firms. Thus, while offering initial insights, this study underscores the need for further inquiry to address these limitations and advance our understanding of the impact of financialization on enterprise behavior and innovation.

## Data availability statement

The data associated with our study been deposited into a publicly available repository. Please see: Tang, Jianxin, 2024, "Financialization and enterprise innovation", https://doi.org/10.7910/DVN/SXCTUA, Harvard Dataverse, DRAFT VERSION.

## Funding

This work was supported by the Hunan Provincial Social Science Achievement Review Committee (No.XSP2023JJZ001), and 10.13039/501100001809National Natural Science Foundation of China (71902038).

## CRediT authorship contribution statement

**Jianxin Tang:** Writing – review & editing, Writing – original draft, Methodology, Conceptualization. **Rizao Gong:** Writing – review & editing, Supervision. **Yun Shi:** Writing – original draft, Validation. **Huilin Wang:** Investigation, Funding acquisition. **Meng Wang:** Validation, Software, Data curation.

## Declaration of competing interest

The authors declare that they have no known competing financial interests or personal relationships that could have appeared to influence the work reported in this paper.
